# Alzheimer’s Disease Neuropathological Change in Aged Non-Primate Mammals

**DOI:** 10.3390/ijms25158118

**Published:** 2024-07-25

**Authors:** Isidro Ferrer

**Affiliations:** 1 Department of Pathology and Experimental Therapeutics, University of Barcelona, carrer Feixa Llarga sn, 08907 Hospitalet de Llobregat, Spain; 8082ifa@gmail.com; 2 Reial Acadèmia de Medicina de Catalunya, carrer del Carme 47, 08001 Barcelona, Spain

**Keywords:** brain aging, Alzheimer, mammals, beta-amyloid, tau, neurofibrillary tangles, cerebral amyloid angiopathy

## Abstract

Human brain aging is characterized by the production and deposition of β-amyloid (Aβ) in the form of senile plaques and cerebral amyloid angiopathy and the intracellular accumulation of hyper-phosphorylated tau (Hp-tau) to form neurofibrillary tangles (NFTs) and dystrophic neurites of senile plaques. The process progresses for years and eventually manifests as cognitive impairment and dementia in a subgroup of aged individuals. Aβ is produced and deposited first in the neocortex in most aged mammals, including humans; it is usually not accompanied by altered behavior and cognitive impairment. Hp-tau is less frequent than Aβ pathology, and NFTs are rare in most mammals. In contrast, NFTs are familiar from middle age onward in humans; NFTs first appear in the paleocortex and selected brain stem nuclei. NFTs precede for decades or years Aβ deposition and correlate with dementia in about 5% of individuals at the age of 65 and 25% at the age of 85. Based on these comparative data, (a) Aβ deposition is the most common Alzheimer’s disease neuropathological change (ADNC) in the brain of aged mammals; (b) Hp-tau is less common, and NFTs are rare in most aged mammals; however, NFTs are the principal cytoskeletal pathology in aged humans; (c) NFT in aged humans starts in selected nuclei of the brain stem and paleocortical brain regions progressing to the most parts of the neocortex and other regions of the telencephalon; (d) human brain aging is unique among mammalian species due to the early appearance and dramatic progression of NFTs from middle age onward, matching with cognitive impairment and dementia in advanced cases; (e) neither mammalian nor human brain aging supports the concept of the amyloid cascade hypothesis.

## 1. Introduction: Human Brain Aging and Cognitive Impairment

Whether Alzheimer’s disease (AD) is a disease unique to humans has been discussed for years [1,2,3,4]. Most formulations have an anthropocentric bias. However, the inquiry is not whether or not AD is a human disease; more appropriately, it is to know whether brain aging differs in humans from other mammals.

Senile plaques (SPs) and neurofibrillary tangles (NFTs) are characteristic lesions in human brain aging and AD. For this reason, these alterations are named Alzheimer’s disease neuropathological change (ADNC). SPs have a central core of β-amyloid (Aβ) surrounded by dystrophic neurites (neuritic plaques: NPs); other Aβ deposits are known as diffuse plaques, which may contain abnormal neural processes but lack dystrophic neurites (diffuse plaques DPs). Aβ deposits are also found in meningeal and parenchymal blood vessel walls, leading to cerebral amyloid angiopathy (CAA). Aβ, which results from the amyloid precursor protein (APP) cleavage by γ- and β-secretases, includes several isoforms of variable length and harboring modifications, such as pyroglutamate modification [5,6,7,8,9,10,11]. Mutations in three genes involved in β-amyloid protein precursor cleavage by γ- and β-secretases, namely *APP*, presenilin 1 (*PSEN1)*, and presenilin 2 (*PSEN2*), cause early-onset familial AD (fAD) [12,13,14,15,16,17,18]. The “amyloid cascade hypothesis” proposed that Aβ causes AD [19,20,21]. Yet, most (about 90% of AD) cases are sporadic and linked to distinct low-penetrance genetic risk factors [22,23,24,25,26,27,28,29,30,31,32,33,34].

NFTs contain hyper-phosphorylated tau (Hp-tau) composed of 3Rtau and 4Rtau isoforms, resulting from alternative splicing of exon 10 of the microtubule-associated protein tau gene (*MAPT*), together with tau acetylation, abnormal conformation, truncation at the C-terminal and N-terminal regions, oligomerization, fibrillization, and aggregation. Pre-tangles form straight filaments of 10 nm, NFT paired-helical filaments (PHFs) with a width between 80 and 20 nm and a cross-over spacing of 80 nm. NFTs but not granular deposits and pre-tangles are argyrophilic with the Gallyas silver method [35,36,37,38,39,40,41,42,43,44,45,46,47,48,49]. Dystrophic neurites and neuropil threads have the same characteristics as NFTs. Tau filaments in NFTs have specific structural folds, as revealed by cryo-electron microscopy [50,51].

The systematic study of hundreds of human brains at different ages served to identify the progression of SPs and NFTs in a random human population. Braak a–c subcortical stages delineate NFTs in selected brain stem nuclei, including the raphe nuclei and locus ceruleus. Braak stages I–II indicate the presence of NFTs in the entorhinal and transentorhinal cortices; stages III–IV indicate the NFT progression to the hippocampus, amygdala, inferior part of the temporal lobe, and limbic system; stages V–VI indicate the NFT progression to the diencephalon and most parts of the telencephalon [52,53,54,55,56,57,58].

SP progression follows consecutive phases involving the neocortex (phase 1), allocortex and limbic system (phase 2), diencephalon and basal nuclei (phase 3), brain stem (phase 4), and cerebellum (phase 5) [59].

NFTs and SPs have different distributions in human brain aging. Aβ deposits first appear in the neocortex, whereas NFTs appear in selected brain stem nuclei and paleocortical regions. Moreover, tau pathology in human brain aging precedes by several decades or years the appearance of Aβ. NFTs affect about 85% of humans at the age of 65 (commonly stages I–IV). About 98% of individuals have NFTs in the telencephalon at 80 at the same NFT stages or more. In contrast, only 30% of people have SPs at 65, and around 60% over 80 [52,53,54,55,56,57,58,60,61]. NFTs without SPs are found in about 35% of individuals older than 90 [58,60].

The prevalence of dementia in humans 65–70 years old is about 1–5% and between 25% and 30% at the age of 85; the majority of cases suffer from dementia of the AD type (AD dementia) [62]. Cognitive impairment and dementia in aged humans with ADNC correlate with NFT pathology rather than with Aβ burden [63].

In 2012, the National Institute on Aging-Alzheimer’s Association (NIA-AA) defined AD as a neurodegenerative disease starting with brain Aβ deposition, followed by NFT pathology [64,65], and clinically categorized it as preclinical AD, mild cognitive impairment (MCI) due to AD, and mild, moderate, and severe Alzheimer’s dementia [66,67,68,69,70,71]. Based on the creed of the β-amyloid cascade hypothesis, the NIA-AA guidelines assumed that the appearance of Aβ is the sine qua non condition for the neuropathological diagnosis of AD. PART (primary age-related tauopathy) was introduced to cover cases with NFT pathology without SPs [72,73]. However, it has been suggested that PART is a part of AD [74]. Alternatively, PART is ordinary in human brain aging, and β-amyloid is later added to produce AD in a time-, rate-, and region-dependent manner [75].

Recently, two complementary hypotheses have been formulated based on the different chronological and regional progression of NFTs and SPs in the human aging brain. One theory states that NFT pathology, progressing according to the Braak stages, is the primary alteration of AD [58]. The other postulates that human brain aging starts early with NFT, followed decades or years later by Aβ pathology as a continuum ranging from a lack of clinical symptoms to devastating dementia [61,76]. Alzheimer’s disease dementia is the most advanced stage of human brain aging, with ADNC occurring in a subset of individuals, whereas in the majority, brain aging with ADNC may be well tolerated or manifest as mild cognitive impairment.

## 2. ADNC in Non-Primate Mammals

Previous studies have analyzed similarities and differences in brain aging and cognitive impairment in humans and non-human primates [77,78]. The present review deals with brain aging in other mammals and explores the implications of differences between humans and other mammals, which eventually make the aged human brain exceptionally vulnerable to neurodegenerative changes [4].

Revised species are summarized in Table 1

**Figure 1:**
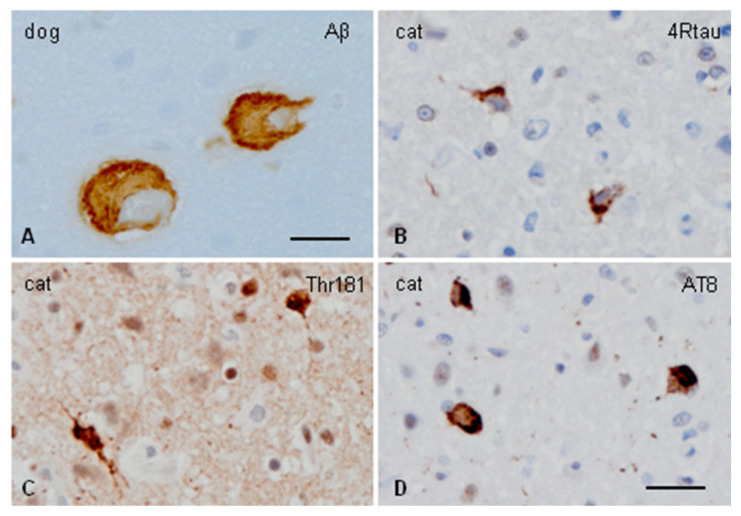
(**A**) CAA in the cerebral cortex of an aged dog (14 years old), and (**B**–**D**) Hp-tau pathology in the cerebral cortex of a cat aged 13 years. Antibodies used recognized total β-amyloid (**A**), 4Rtau (**B**), Hp-tau Thr181 (**C**), and Hp-tau Ser 202/Thr205 (clone AT8). Bar in (**A**) = 80 microns; (**B**–**D**) bar in (**D**) = 40 microns.

CAA and tau pathology in aged dogs and cats is illustrated in Figure 1

### 2.1. Pinnipeds

Neuritic plaques and CAA in the frontal cortex, and fibrilar aggregates of Hp-tau containing 3R and 4R isoforms, composed of straight filaments 10 µm in diameter, in neurons and dystrophic neurites were observed in the frontal cortex and one case in the hippocampus, in aged pinnipeds including sea lions (*Zalophus californianus*), seals, and walruses (*Odobemus rosmarus*) [79]. In sea lions, all SPs and most CAA lesions were positive for Aβ42, Aβ43, and Aβ40, while capillary CAA lesions were negative for Aβ40 [80].

### 2.2. Bears

Diffuse plaques accompanied by neurofilament-immunoreactive but tau-negative processes were found in an aged polar bear (*Ursus maritimus*); Ph-tau and NFTs were not identified [81]. Another study described the presence of diffuse plaques of Aβ42/43, but not Aβ40, and CAA immunolabelled with anti-Aβ42/43 and Aβ40 antibodies. PHF-1-positive NFTs were also reported in aged polar bears [82]. Finally, the neuropathological study of two polar bears aged 28 and 37 (life span of 15–18 years; in captivity, about 30) with abnormal behavior showed neuritic plaques revealed with Bielschowsky silver stains and numerous Aβ plaques in the neocortex, allocortex, striatum, and cerebellum. Both bears had extensive CAA. A silver-stained hippocampus revealed neurons described as NFTs [83].

An aged Asian brown bear (*Ursus arctos*) had NFT-like deposits composed of straight 10–16 nm filaments that were immunoreactive with antibodies against tau and PHF antibodies [81]. Numerous senile plaques and CAA were seen in the brain of an American black bear (*Euarctos ursus americanus*) over 20 years old [84].

### 2.3. Dogs (Canis lupus familiaris)

Diffuse plaques, perivascular amyloid plaques, and CAA, mainly localized in the cerebral cortex and rarely in the hippocampus and striatum, are currently seen in dogs aged more than 8–10 years (life span of about 15 years depending on the species) [85,86,87,88,89,90,91,92,93,94]. Aβ42/43 predominates in diffuse plaques; Aβ42/43 and Aβ40 occur in CAA [95,96]. Another study reports that most CCA and primitive SPs are positive for Aβ42, Aβ43, and Aβ40; however, diffuse SPs and capillary CAA lesions are negative for Aβ40 [80]. Canine plaques also contain epitopes Aβ1-17, Aβ17-24, and Aβ1-28 [95]. Aβ deposition in plaques and CAA is highly heterogeneous in dogs from different breeds and sizes [97]. Pyroglutamate at the third residue (pyroGlu-3 Aβ) has been identified in beagle dogs [98]. Aβ phosphorylation was reported in another study [99]. Aβ deposition in the aged canine frontal cortex begins with diffuse deposits in the deep cortical layers, followed by the development of deposits in the outer layers [100].

Canine cognitive dysfunction (CCD) is common in aged (>8 years) dogs, affecting between 14% and 35% of the pet dog population. The clinical symptoms consist of confusion, anxiety, disturbance of the sleep/wake cycle, and decreased interaction with owners [101]. Aβ plaque density correlates with age but not cognitive impairment [102]. However, another study revealed a strong association between Aβ deposition and deficits in discrimination learning and reversal learning but not in other tasks; NFTs were absent in the same canine series [103]. Such correlation was further addressed in another series showing a significant association when correcting for age between Aβ plaque density in the prefrontal cortex and hippocampus/entorhinal cortex, but not in the temporal cortex, and CCD in old dogs [104].

Most studies have pointed to the absence of NFTs in aged dogs. However, small numbers of AT8-immunoreactive neuronal deposits have been detected in the hippocampus in aged dogs [91,94,95]. Moreover, the Hp-tau Ser396 antibody, which recognizes early Hp-tau deposits, stains neurons in the parietal cortex and hippocampus in one study [105] and throughout the limbic system in another [106]. The presence of Aβ42 oligomers and Ph-tau in the hippocampus correlates with cognitive impairment [105]. Another study indicates that Hp-tau in the cerebral cortex and limbic system correlates with CCD [94].

### 2.4. Domestic and Wild Cats

Diffuse plaques were detected in aged domestic cats (*Felis catus*) (17–21 years old) and distributed throughout the cortical layers of the parietal lobes (life span of 12–18 years; in captivity, 25). CAA and diffuse plaques were stained with the antibody Aβ42 but not Aβ40 [80,95,107]. Similar results were obtained in another cohort [108]. Diffuse plaques, stained with Aβ42 and Aβ17-24 antibodies but not with antibodies directed to Aβ40 and N-terminal Aβ, were noticed in aged (16–21) but not in young (<4 years) cats [108]. In addition, Hp-tau pre-tangles were found in the hilus of the hippocampus in two aged cats [108].

Aged cats may suffer from cognitive dysfunction syndrome (CDS), characterized by behavioral abnormalities, including excessive vocalization, increased affection or attention with owners, altered sleep–wake cycles, house-soiling, spatial and temporal disorientation, alterations in activity, anxiety, and learning and memory deficits [109]. Studies have been designed to determine a possible correlation between CDS and Aβ and Hp-tau pathology in elderly cats.

Brain tissue from 19 domestic cats was assessed, 17 of which had clinical signs of CDS. Extracellular Aβ immunoreactivity was observed in seven cats over ten years, and neuronal Ph-tau immunostaining was observed in two cats aged 11 and 13. However, no NFTs were detected. These observations suggest that neurological dysfunction in aged cats is not universally correlated with Aβ and even lesser with Hp-tau pathology [110]. Similar results were obtained in the study of 55 cats [111]. Aβ-immunoreactive diffuse plaques were present in the cerebral cortex, extending to the hippocampus in some animals; intraneuronal Aβ deposits were also observed in young but not in old cats. Hp-tau pre-tangles were found in the cerebral cortex and lesser in other brain regions, including the entorhinal cortex and hippocampus; intranuclear tau was found in young but not in aged cats. Ten cats had CDS, but no correlation was found between Aβ and Ph-tau pathology and cognitive impairment in aged cats [111]. β-amyloid diffuse plaques, predominantly in cortical layers IV and VI, were found in 27 of the 32 aged cats used in another study; neuritic plaques were not found. Only 4 of the 27 cases had Hp-tau pre-tangles, with neuropil threads restricted to the entorhinal cortex in 3 and involving the entorhinal cortex, hippocampus, and cerebral neocortex in 1 [112].

Aβ deposits were observed in 13 among 22 captive cheetahs (*Acinonyx jubatus*); neuronal Hp-tau in the form of pre-tangles and NFTs also occurred in the parahippocampal cortex and CA1 region of the hippocampus in 10 of the cheetahs with Aβ deposits. Two cheetahs with the most severe abnormal Hp-tau immunoreactivity showed clinical cognitive dysfunction [113].

Granular deposits containing Aβ42 but not the N-terminal of human Aβ were found in the cerebral cortex of six wild Tsushima leopard cats (*Prionailurus bengalensis euptilurus*) that live exclusively on Tsushima Island, Japan; neuritic plaques were absent [114]. Interestingly, analysis of the leopard cat APP gene detected a base substitution, which altered the N-terminal amino acid sequence of the Aβ protein.

In addition, pre-tangles and NFTs were seen in five of the six leopard cats with Aβ deposits localized in the parahippocampal gyrus, spreading to the hippocampus and ectosylvian gyrus in the more severely affected cats. Ultrastructurally, Hp-tau deposits were composed of straight filaments and filaments consistent with paired structures 10–20 nm in diameter. Some oligodendrocytes also contained aggregates of Hp-tau [114].

### 2.5. Wolverine

Diffuse and neuritic plaques stained with anti-Aβ antibodies and neuritic plaques also identified with Congo red and thioflavin S were found in the brain of an aged (over 14 years old) male wolverine (*Gulo gulo*). CAA also occurs in the meningeal and cerebral blood vessels. Intracellular argyrophilic NFTs, immunoreactive with anti-Hp-tau (AT8) antibodies, were observed in the parahippocampal gyrus, CA1 region of the hippocampus, and cerebral cortex. Many hippocampal neurons had granulovacuolar degeneration. Microhemorrhages or small confluent hemorrhagic regions were present within the cerebral cortex, many closely associated with CAA [115].

### 2.6. Cetacea

Neuritic plaques and deposits of Hp-tau in neurons, neuropil threads, and dystrophic neurites were found in three species of oceanic dolphins, but only Hp-tau pathology and no β-amyloid plaques in one animal. The series included two Risso’s dolphins (*Grampus griseus*), five long-finned pilot whales (*Globicephala melas*), five white-beaked dolphins (*Lagenorhynchus albirostris*), five harbor porpoises (*Phocoena phocoena*), and a single bottlenose dolphin (Tursiops truncatus): age unknown [116].

Aβ plaques and granular Hp-tau deposits were found in six of nine cetaceans, including five deep-diver animals, one Cuvier’s beaked whale (*Ziphius cavirostris*), two Blainville’s beaked whales (*Mesoplodon densirostris*), one short-finned pilot whale (*Globicephala macrorhynchus*), one Risso’s dolphin (*Grampus griseus*), four shallow-divers, three Atlantic spotted dolphins (*Stenella frontalis*), and one captive neonatal bottlenose dolphin (*Tursiops*). Interestingly, Aβ and Hp-tau pathology was higher in deep-diver animals, thus suggesting that this subgroup of cetaceans is more vulnerable to sustained and repetitive brain hypoxia [117]. No pathology was found in the neonatal bottlenose dolphin. However, Aβ plaques and Hp-tau pathology were identified in one captive 40-year-old bottlenose dolphin (*Tursiops truncatus*) [118].

Increased numbers of Aβ plaques and dystrophic neurites were observed in the auditory cortex compared to the visual cortex and brainstem, and high levels of cyanobacterial neurotoxin β-methylamino-L-alanine (BMAA) in 13 of 14 stranded dolphins in Florida and Massachusetts, thus suggesting the potential impact of cyanotoxin exposure and AD-like pathology [119]. Increased BMAA levels occurred in parallel with increased methylmercury (MeHg), a synergistic neurotoxicant with BMAA; there was a 3-fold increase in gene transcription related to Aβ plaques, NFTs, neuritic plaques, and TDP-43 intracytoplasmic inclusions, and up to a 14-fold increase in AD-type neuropathology was identified in affected dolphins [120]. Aβ plaques, NFTs, granulovacuolar degeneration, and Hirano bodies were present in the hippocampus; there were TDP-43 cytoplasmic inclusions in neurons throughout the cerebral cortex, midbrain, and brainstem; and P62/sequestosome-1 was observed in the amygdala, hippocampus, and frontal cortex in a beached harbor porpoise (*Phocoena phocoena*) exposed to BMAA [121].

### 2.7. Cattle

Intracellular and extracellular Aβ deposition has been detected in aged cattle’s cerebral cortex, hippocampus, and cerebellum (age unknown; life span of about 20 years for domestic cattle). Aβ comprises C-terminal truncated forms but does not form fibrillar aggregates, thus suggesting that cattle are protected from developing mature plaques [122]. However, another study reported diffuse plaques, CAA, and intracellular Aβ deposition in aged cattle. Moreover, cow-derived Aβ aggregates accelerated Aβ deposition in the brain of AD transgenic animals [123].

### 2.8. Sheep

In contrast with other species, NFT-like structures and clusters of degenerating neurites stained by silver impregnation and thioflavin-S and immunoreactive with antibodies against microtubule-associated protein tau occur in aged sheep aged 8–14 years (life span of 18–20 years) (*Ovis aries*); tau-immunoreactive tangles are composed of typical PHFs [124]. Subsequent studies demonstrate that Hp-tau is localized at dendritic branched points associated with clusters of ribosomes at early stages of NFT formation [125]. SPs are also found in the brains of aged sheep [126].

### 2.9. Equids

Diffuse β-amyloid plaques were found in the parietal cortex in nine of thirteen aged donkeys (*Equus africanus asinus*) (˃30 years; life span of 27–40 years), and NFT-like deposits were found, as revealed with the AT8 antibody, in seven with a predominance in the cortical areas. Two donkeys also showed NFT-like pathology in the hippocampus [127].

### 2.10. Guinea Pigs (Cavia porcellus)

Diffuse deposits of Aβ were found in the hippocampus of old guinea pigs aged more than 4 years (life span of 5–7 years); total tau, as revealed with the antibody Tau-5, is increased in the cytoplasm of neurons, but evidence of NFTs and Hp-tau deposits is absent [128].

ADNC was also assessed in two non-transgenic guinea pig strains, the standard PigmEnTed and Dunkin-Hartley strain [129]. In addition to Aβ42-immunoreactive diffuse plaques in the hippocampus, hippocampal neurons contained Hp-tauThr181; yet, the low magnification of the images and the lack of detailed information do not permit further evaluation of the Ph-tau deposits in these animals [129].

### 2.11. Degus

Wild-aged *Octodon degu* may develop SPs, CAA, and tau pathology. These changes are more severe in animals with altered burrowing behavior [130,131,132,133]. The amino acid homology (97.5%) of Aβ sequences in *O. degu* and humans is a significant factor in the appearance of Aβ plaques in this rodent [130]. However, these results are not reproduced in other series of animals in captivity [134,135], thus advocating that natural factors are involved in developing ADNC in aged *O. degu*. In a cognitively impaired subset of aged, outbred degus, numerous Aβ plaques are stained with antibodies anti-Aβ40, A42, Aβ43, and pyroglutamate AβpE3 [136]. Some show a ribbon decorated with anti-tau antibodies in adjacent sections, thus mimicking dystrophic neurites [136]. Regarding abnormal tau deposits in degus, the images of the distinct papers are challenging to interpret due to the low magnification and the lack of definite tau patterns using various anti-tau antibodies, including human tau, tau amyloid sequences, and AT8 and PHF1 antibodies [130,132,136].

### 2.12. Rabbits

Aged rabbits do not develop ADNC, but cholesterol-enriched diets, more effectively if accompanied by trace amounts of copper, generate Aβ-immunoreactive extracellular deposits and CAA in adult New Zealand white rabbits [137,138,139,140]. Aβ peptide-bearing amino-terminal pyroglutamate at position 3 AβN3 (pE) is also observed in blood vessels in cholesterol-enriched diet-fed rabbit brains [141]. Hp-tau deposits are absent [139].

### 2.13. Tree Shrews

Aged Chinese tree shrews (*Tupaia belangeri chinensis*), six years or older (live span of 2–3 years, but in captivity, up to 12 years), develop an impaired cognitive performance in the hole-board test and novel object recognition compared to the adult tree shrews. Moreover, aged tree shrews show Aβ17-24- immunoreactive plaques in the hippocampus and cerebral cortex, and granular accumulation of Hp-tau AT8 and Hp-tau Thr231 in neurons of the dentate gyrus and hippocampal subfields; pre-tangles and NFTs are absent [142]. These observations align with earlier descriptions showing Aβ- and APP-immunoreactive structures, referred to as senile plaque-like structures in the brains of aged tree shrews [143,144].

## 3. Summary of ADNC and Abnormal Behavior in Aged Mammals

ADNC in non-primate mammals is outlined in Table 2.

Aged wild mice and rats do not have ADNC, although transgenic mice bearing human mutations in the APP-related genes and MAPT develop Aβ plaques, CAA, and Hp-tau intracytoplasmic aggregates. Similarly, aged wild rabbits do not have ADNC, although diets enriched in cholesterol plus copper may induce ADNC pathology. All the other revised mammalian species may develop naturally Aβ diffuse plaques, most CAA, and a few NP in old age. Aβ deposition is primarily neocortical, with predominance in the frontal and parietal lobes, followed in some cases by the hippocampus and limbic system. Thus, Aβ pathology in aged pinnipeds, bears, dogs, cats, wolverines, odontocetes, calves, sheep, donkeys, tree shrews, and subpopulations of degus is similar to Aβ pathology in aged humans notwithstanding that Aβ pathology is more severe in humans than in other mammals.

Hp-tau pathology is less frequent and often restricted to a few neurons with intracytoplasmic deposits in pinnipeds, bears, dogs, wolverines, cetaceans, sheep, donkeys, and tree shrews. Scanty NFTs involving the cerebral cortex and hippocampus are found in bears, wolverines, cetaceans, and sheep. Currently, Hp-tau neuronal deposits in mammals are granular or diffuse, consistent with pre-tangles. NFTs are less common in aged mammals. Accumulating Hp-tau in aged mammals is challenging to fit with common human tauopathies [145].

Distinguishing abnormal behavior is problematic in wild animals, except for stranded cetaceans. Altered behavior has not been reported in domestic-aged cattle, sheep, and donkeys. However, a correlation between cognitive deficits and ADNC has been described in tree shrews and two polar bears. Altered burrowing behavior is accompanied by increased Aβ deposits and unclassifiable tau pathology in aged *Octodon degu’s* subgroups.

More information is available on aged dogs and cats. Canine cognitive dysfunction (CCD) has been associated with increased Aβ burden, Aβ42 oligomers and Hp-tau in the hippocampus, and Hp-tau in the cerebral cortex and limbic system.

Cognitive dysfunction syndrome (CDS) occurs in aged cats. However, several series of aged cats found no correlation between Aβ deposits and even less with Hp-tau and CDS.

## 4. Comparative Brain Aging ADNC in Mammals and Humans

Aβ deposition in the form of SPs and CAA is a common alteration in most aged mammals’ brains. Diffuse plaques predominate in non-primate mammals, non-human primates [77,78], and human’s early Aβ deposition stages. In most mammals, DPs appear in the frontal and parietal cortices and rarely progress to other cortical areas and diencephalic regions. NPs, when present, occur at more advanced stages. In contrast, NPs predominate in the aged human brain. Aβ deposits progress from the neocortex, allocortex, and limbic system, diencephalon and basal nuclei, brain stem, and cerebellum following Thal’s phases [59]. The neocortex is the most recent structure in brain phylogeny and typically identifies the mammalian brain evolution from the reptilian brain. Since Aβ manufacturing occurs at the membranes, it can be inferred that senescent neural membranes, including those of the cerebral blood vessel walls, favor Aβ production.

The consequences of Aβ pathology in the aged brain on cognition are very discrete in most mammals. There is no apparent altered behavior; if present, no correlation is established between cognitive impairment and Aβ pathology in most species.

Hp-tau pathology is much more discrete in aged mammals, including non-human primates [77,78], than Aβ deposition. If present, Hp-tau occurs in the form of scanty intracellular granular deposits and pre-tangles. NFTs are rare, and except for a few cases, they do not have the typical distribution of human NFTs in aging. In contrast, NFT pathology is a characteristic trait of human brain aging that appears at early ages and progresses, with individual variations, from selected nuclei of the brain stem and the entorhinal and transentorhinal cortices to the hippocampus and limbic system, and most parts of the cerebral cortex and diencephalic nuclei [52,53,54,55,56,57,58]. Studies with cryo-electron microscopy would be helpful to identify tau folds in aged mammals.

NFT pathology reflects a particular vulnerability of the human cytoskeleton to aging compared with other species. NFT pathology progresses exponentially with age from the sixties onward in modern humans, leading to dementia of the AD type in a subgroup of individuals, which represents about 1–5% at 65 and 25–30% at the age of 85 [62].

Comparative neuropathology of brain aging further confirms the misconception formulated by the β-amyloid cascade hypothesis. Aβ deposition and Hp-tau pathology are different processes that may converge in the aged brain.

The need for uniformity in the methodology of neuropathological studies in mammals is a matter of concern; lack of uniformity makes it difficult to compare lesions in the same regions in different species. For this reason, Appendix A provides a proposal of suggested regions and histological methods.

## Figures and Tables

**Table 1:** Aged mammals included in this review.

Carnivora	Carnifornia, Pinnipedia	pinnipeds	sea lions, seals, walruses
	Carnifornia, Ursidae	bears	polar bear, Asian brown bear, American black bear
	Cannidae	dogs	
	Felidae, Felinae	cats	domestic cats, cheetahs, Tsushima leopard cats
	Mustelidae	wolverine	wolverine
Artiodactyla	Cetacea	cetacea	Risso’s dolphins, long-finned pilot whales, white-beaked dolphins, harbor porpoises, bottlenose dolphin, Cuvier’s beaked whale, Blainville’s beaked whales, short-finned pilot whale, Atlantic spotted dolphins, bottlenose dolphin
	Bovidae, Bovinae	cattle	cattle
	Bovidae, Caprinae	sheep	sheep
Perissodactyla	Equidae	donkey	donkey
Rodentia	Cavidae	guinea-pig	guinea
	Octodontidae	degus	degus
Lagomorpha		rabbits	rabbit
Euarchonta	Scadentia	three shrew	three shrew

**Table 2:** Summary of Aβ and tau deposits in aged mammals. DP: diffuse plaques; NP: neuritic plaques; CAA: cerebral amyloid angiopathy; Hp-tau: hyper-phosphorylated tau; NFT: neurofibrillary tangles; nd: not described. Semiquantitative signs are only approximate as the studies were carried out by different authors, using different techniques, and according to subjective estimations of the changes; they express the maximal rate reported in every species. BMAA: β-methylamino-L-alanine.

Species	DP	NP	CAA	Hp-tau	NFT	Comments
pinnipeds	+	+	+	+	0	
bears	++	++	+	+	+	
dogs	+	0	+	+	0	
cats	++	0	+	+	0	NFT+: cheethas
wolverine	++	+	+	++	+	*n* = 1
cetacea	+	+	nd	+	+	higher in animals exposed to BMAA
cattle	+	0	nd	0	0	
sheep	+	0	nd	+	+	
donkey	+	0	nd	+	0	
guinea-pig	+	0	0	0	0	
degus	+	0	0	+ (?)	0	depending on environmental factors
rabbits	0	0	0	0	0	DP in cholesterol-fed + cooper
three shrew	+	0	0	+	0	

## Appendix A. Suggested Sampling and Staining for Age-Related Neurodegenerative Diseases in Veterinary Neuropathology

Braak stages for NFT and amyloid pathology and Thal’s phases for β-amyloid deposition were obtained from the systematic study of selected brain regions in humans, and they are well established in categorizing human brain aging and Alzheimer’s disease. However, no similar standardized method and classification are currently used to study brain aging in different species. Moreover, stages for human cases may not adapt to other mammals.

The following sections are recommended in the neuropathological study of neurodegenerative diseases of human brain samples [146]: neocortical areas: middle frontal, inferior and superior parietal, superior, middle and inferior temporal, precentral, anterior and posterior cingulate, insular cortex, and occipital; allocortical areas: olfactory bulb and tract, piriform cortex, hippocampus, and entorhinal cortex; subcortical nuclei: caudate, putamem, accumbens, pallidum, thalamus, subthalamus, hypothalamus, mammillary bodies, amygdala; brain stem and cerebellum: midbrain with red nucleus and substantia nigra, pons with locus coeruleus, medulla oblongata with dorsal motor nucleus of the vagus nerve and inferior olives, vermis of cerebellum and hemisphere with dentate nucleus; spinal cord at cervical, dorsal, lumbar and sacral levels; spinal ganglia; anterior and posterior roots. Large hemispheric blocks are recommended.

Similar regions would be suitable in the neuropathological study of other mammals, although sample limitation is standard in routine practice. Yet, several areas can be assessed in the same tissue section in many small mammals.

Regarding staining methods, hematoxylin and eosin and Nissl (or Klüver-Barrera) staining is routine in many laboratories; methenamine silver (for amyloid plaques), Gallyas (for fibrillary tau deposits), and thioflavin S (or Congo red) demonstrate the presence of amyloid fibrils. Regarding immunohistochemistry, total Aβ, Aβ_1–40_, Aβ_1–42_, Aβ_17–40_, and antibodies against pyroglutamate-modified N-terminal Aβ truncated peptides are helpful to characterize Aβ deposits.

Tau deposits can be recognized with total anti-tau, anti-3R, and anti-4R antibodies and further discriminated based on their recognition with various phospho-specific anti-Hp-tau antibodies. Positivity with specific ant-Hp antibodies identifies the state of complexity of tau hyper-phosphorylation: one single site: Thr181, Srr199, Ser231, Ser262, Ser396, and Ser422; two phosphorylation sites: clone AT8 (Ser202, Thr205) and PHF (Ser119, Ser202); truncated tau at aspartic acid 421 (tau-C3); tau conformational changes Alz50, MC-1, and tau oligomers; nitrated tau [147].

It is worth mentioning that tau phosphorylation in mammals’ brains has been less extensively studied than in humans. Therefore, tau-hyperphosphorylation may involve phosphorylation sites divergent in humans and other mammals. In the same line, tau folds, identified with cryo-electron microscopy, have not been assessed in mammals other than human neurodegenerative diseases.

Finally, other antibodies are helpful in the study of neurodegenerative diseases in aged individuals, including those raised against ubiquitin, p62, phosphorylated neurofilaments, α-synuclein, TDP-43, phospho-TDP-43, glial fibrillary acidic protein (GFAP), and Iba1. Further markers may be used when needed.
